# Composite endpoints for malaria case-management: not simplifying the picture?

**DOI:** 10.1186/1475-2875-13-494

**Published:** 2014-12-13

**Authors:** Matthew E Cairns, Baptiste Leurent, Paul J Milligan

**Affiliations:** MRC Tropical Epidemiology Group, London School of Hygiene and Tropical Medicine, Keppel Street, London, WC1E 7HT UK

## Abstract

Rapid diagnostic tests (RDTs) for infection with *Plasmodium spp.* offer two main potential advantages related to malaria treatment: 1) ensuring that individuals with malaria are promptly treated with an effective artemisinin-based combination therapy, and 2) ensuring that individuals without malaria do not receive an anti-malarial they do not need (and instead receive a more appropriate treatment). Some studies of the impact of RDTs on malaria case management have combined these two different successes into a binary outcome describing ‘correct management’. However combining correct management of positives and negatives into a single summary measure can be misleading. The problems, which are analogous to those encountered in the evaluation of diagnostic tests, can largely be avoided if data for patients with and without malaria are presented and analysed separately. Where a combined metric is necessary, then one of the established approaches to summarise the performance of diagnostic tests could be considered, although these are not without their limitations. Two graphical approaches to help understand case management performance are illustrated.

## Background

Case management of malaria has been revolutionised by the introduction of rapid diagnostic tests (RDTs), which allow parasitological diagnosis at the point of care in a much wider range of settings than was previously possible [1]. Diagnostic testing is the first stage of the ‘T3: Test. Treat. Track.’ approach recommended by WHO [2]. The potential advantages of wider availability and higher quality of diagnostics are two-fold: firstly, increasing the percentage of those with malaria who receive prompt and effective treatment with an artemisinin-based combination therapy (ACT), and secondly, increasing the percentage of those without malaria who do not receive an antimalarial and instead are managed appropriately according to their condition [3]. Withholding treatment from RDT-negative patients should avoid unnecessary side effects, avoid wasted drugs, and help reduce selection pressure for resistance to antimalarials used for case management [3], although some of these benefits have been challenged in certain situations [4]. A further benefit of identifying malaria-negative cases is that this may help determine the true cause of fever, which could lead to better clinical outcomes. However, a recent study in Tanzania showed that 70% of patients presenting with fever had a viral illness as the underlying cause [5]; there may be limited scope for improved clinical outcomes where there is no obvious treatment beyond management of symptoms. Over-use of antibiotics among those who test negative for malaria is also a concern [6]. Guidelines for the evaluation of diagnostic procedures emphasise the importance of measuring clinical benefit [7, 8] but in practice evaluation has often relied on assessment of sensitivity, specificity and predictive values [7].

To address these issues, a number of studies have looked at the impact of RDTs on adherence to the current guidelines for malaria treatment [3]. In some of these studies the two different successes (treatment of positives and non-treatment of negatives) have been combined into a composite outcome describing the overall success rate of ‘correctly managed’ patients, either for descriptive purposes or to create a binary outcome that is used for formal analyses. This appears to have been done for two main reasons. Firstly, to provide a simple, summary measure of case-management success, which might seem appealing when comparing different diagnostic strategies, or in a randomised trial where defining a unique primary outcome is usually recommended. For example, a composite outcome has been used to describe the situation in cross-sectional studies prior to RDTs being introduced [9] or following introduction of RDTs [10], to compare case management following an intervention to improve management of febrile patients [11] and to monitor changes in case-management over time [12]. Secondly, in many settings the proportion of patients with malaria is low, and therefore when looking at factors associated with correct management of parasitaemic patients, there are an insufficient number of positives to allow a meaningful analysis of risk factors. Combining the positives with the larger number of negatives appears to have been undertaken to provide a larger sample size for multivariable analysis [9–11]. However, as shown below, combining correct management of positives and negatives at best provides an ‘average’ that is hard to compare with other settings, and at worst distorts the true situation. Separate presentation and analysis of positives and negatives would avoid these issues.

### Problems with a composite outcome of case-management success

A number of the issues related to combining all correctly managed patients together are analogous with well-established theory related to diagnostic tests: sensitivity and specificity are routinely reported as inherent characteristics of a test, rather than the overall percent correctly diagnosed, because for a particular test, the percent correctly diagnosed will depend on the relative number of positives and negatives that take the test. Likewise, the overall percent of patients that are correctly managed in a particular scenario (the combined management rate, CMR) depends on 1) the percentage of true positives who are successfully managed (positive management rate, PMR, analogous to sensitivity), 2) the percentage of true negatives who are successfully managed (negative management rate, NMR, analogous to specificity), and 3) the relative frequency of positives and negatives who must be managed (the prevalence among those seeking treatment) (Table 1). Note that these are not true rates but proportions. However, unless the PMR and NMR are identical, then the CMR will vary as a function of prevalence, being equal to the NMR at zero prevalence and equal to the PMR in the (hypothetical) situation of 100% prevalence. Comparisons of the CMR between scenarios (over time for a particular location, or between locations) are only straightforward to interpret if prevalence is the same, and if one of the two management rates remains constant. In scenarios with low prevalence (which will be found far more commonly than prevalence close to 100%), the management of parasite negative individuals will dominate the overall combined management rate, and could mask genuine improvements (or deterioration) in the appropriate management of parasitaemic patients.

**Table 1:** **Measures of case management success**

		True status		
How managed		Positive	Negative	Total
	Treated	a	c	a+c
	Not treated	b	d	b+d
	Total	a+b	c+d	N
Prevalence		(a+b)/N		
Positive management rate (PMR)		a/(a+b)		
Negative management rate (NMR)		d/(c+d)		
Combined management rate (CMR)		(a+d)/N		**OR**
		PMR*p+NMR*(1-p)		
**Predictive values**				
% positive among treated		a/(a+c)		
% negative among not treated		d/(b+d)		

Furthermore, combining management of malaria positives and malaria negatives together implicitly gives equal weight to each of these (and equal weight to the corresponding failure to manage correctly). The relative importance of correctly managing both types of patient is likely to differ depending on the application (e.g. treating acutely ill patients versus mass-screening and treatment of asymptomatics to reduce transmission), but arguably, failure to treat a patient with malaria is usually more serious than inadvertently providing an ACT to a malaria negative patient. Methods from the diagnostic literature exist that would allow this weighting to be altered, in the same way that sensitivity or specificity could be considered as more important for a diagnostic test [13], or performance of a test weighted by utility gains and losses [14]. However, the choice of weights is subjective. Description and analysis of positives and negatives separately avoids having to make this judgement, leaving this to the interpretation stage, and allows comparison between settings without having to formalise the weighting.

Finally, regression of combined correct management as a binary outcome could mask factors that are associated with correct treatment of positives, particularly if prevalence among those seeking care is low. For example, a hypothetical risk factor that is strongly associated with correct management of positives (an odds ratio of approximately 3) if observed among 100 positives, would not be identified as being associated when combined with 900 negatives, if there is no association of the risk factor with correct management of negatives (Table 2). It will also be misleading if the factor is differentially associated with the PMR and NMR, as might be the case for factors such as ACT availability (low stock might lead to highly selective use (low PMR, high NMR), and vice versa). Whether the association between factors and correct management differs according to the malaria status can formally be investigated using tests for interaction (effect modification). However, these tests are not always performed, and typically have low power. In addition, if the PMR and NMR differ, then because the CMR will change with malaria prevalence as described above, any factors that are themselves associated with prevalence (e.g. age), will appear associated with the CMR.

**Table 2:** **Hypothetical result showing masking of risk factor in combined analysis**

	True status			
	Malaria (N = 100)		No malaria (N = 900)	
	Exposed	Unexposed	Exposed	Unexposed
Correct management	37	25	225	225
Incorrect management	13	25	225	225
**Total**	50	50	450	450
Stratified odds ratio	2.85 (1.23, 6.60), p = 0.015		1.00 (0.77, 1.30) p = 1.0	
Combined OR	1.10 (0.86, 1.41), p = 0.45			

### Composite outcomes from the diagnostic literature

The literature on evaluation of diagnostic tests recommends against using the overall accuracy to compare tests (e.g. [13, 15]). For meta-analyses, bivariate approaches can account for sensitivity and specificity simultaneously [16]. If a single measure is desired for summary purposes, a number of options exist (Table 3) but each has its limitations. In the definitions that follow, to illustrate the example of case management considered here, positive management rate has been substituted for sensitivity, and the negative management rate for specificity.

**Table 3:** **Combined measures of overall case management success**

Measures of overall success	Formula
Youdens index	PMR+NMR - 1
Likelihood ratio for positives (LR^+^)	PMR/[1 – NMR]
Likelihood ratio for negatives (LR^-^)	[1 – PMR]/NMR
Diagnostic Odds ratio	[ PMR/(1-PMR)]/[(1-NMR)/NMR]

PMR: positive management rate; NMR: negative management rate.

Youden’s index [17] combines PMR and NMR as (Y = PMR + NMR - 1), which is not affected by prevalence, but has the problem that different combinations of PMR and NMR can give the same value of the index (i.e. the index is degenerate, and thus when presented alone is not very informative). The diagnostic odds ratio (DOR) has also been suggested as a summary measure, this is calculated as [PMR/(1-PMR)] / [(1-NMR)/NMR] i.e. the odds of receiving an ACT among those with malaria, relative to the odds of receiving an ACT among those without malaria. Alternatively, the DOR can be interpreted as the odds of malaria among those treated, relative to the odds among those not treated. The DOR does not depend on the prevalence but a drawback is that this index is also degenerate [15]. Further, if the 2 × 2 table contains zeroes (for example if all positives are managed appropriately), the DOR will be undefined. Alternatively, the ratio of the true-positive rate to the false-positive rate (likelihood ratio for positives, LR^+^ = PMR/[1–NMR]) and the false negative rate to the true negative rate (likelihood ratio for negatives LR^-^ = [1-PMR]/NMR) can be calculated. Higher values of LR^+^ indicate better discrimination, as a higher proportion of those treated were actually infected. Conversely, smaller values of LR^-^ indicate better discrimination, because they show that fewer of those not treated were not infected. LR^+^ and LR^-^ do not depend on the prevalence of the disease. Note that the DOR is the ratio LR+/LR-.

**Figure 1:**
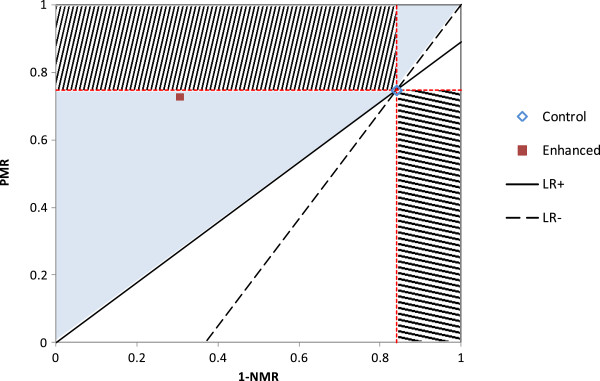
**Biggerstaff method for comparing case-management strategies.** The blue diamond shows the positive management rate plotted against 1- negative management rate for the control group in [19]. The red square shows the estimate of PMR and 1- NMR for the enhanced training group. Dotted red lines show equivalent PMR and NMR. The upper left rectangle (upward shading) shows the region where a strategy would be considered superior both in term of NMR and PMR; the lower right quadrant (downward shading) shows the region where a new strategy would be considered inferior in terms of NMR and PMR. The solid black line shows the line of constant likelihood ratio for positives, and the dashed black line shows the line of constant likelihood ratio for negatives. The blue shaded area shows the region where an alternative strategy would be considered superior on the basis of higher LR+ and lower LR-, as discussed in Biggerstaff [18].

Biggerstaff [18] describes a graphical approach to compare diagnostic tests using the likelihood ratios, which could be used to compare different case-management strategies. An example using data from a recent trial to improve adherence to malaria treatment guidelines [19] is shown in Figure 1. In the control group, 208/278 patients with malaria received an ACT (PMR = 74.8%); but only 38/239 negative patients did not receive an ACT (NMR = 15.9%). In the group given enhanced training, 363/498 patients with malaria received an ACT (PMR = 72.9%), and 527/759 negatives were not treated (NMR = 69.4%). Thus the main change was an improvement in the management of negatives, at the cost of a slight fall in the correct management of positives. When a new strategy falls in the upper left quadrant or lower right quadrant (as defined by the lines of equivalent PMR and NMR to the reference strategy), the new strategy is clearly superior or clearly inferior, respectively. When a new strategy falls into the bottom left or upper right quadrant, whether this is preferred will depend on how much of a decrease in NMR or PMR can be compensated by an increase in the other rate. According to the method proposed by Biggerstaff, any combination of PMR and NMR that lies above the lines for the LR^+^ and LR^-^ (the area shaded blue in Figure 1) would be considered ‘superior’ on the basis of the likelihood ratios. However, a case management strategy with a higher LR^+^ and lower LR^-^ will not systematically be preferred, because different importance may be attached to failure to treat positives, or the incorrect treatment of negatives.

Newcombe [13] describes a graphical approach that allows the relative importance of the two types of incorrect diagnosis to be specified in a simultaneous comparison of sensitivity and specificity between two tests, (or as applied to the current example, simultaneous comparison of case management of positives and negatives between two different management strategies). This is done by plotting the difference in the probability of correct management between the two strategies as a function of the prevalence, with a confidence interval. The case management strategy being evaluated can be considered superior to the control where this interval lies entirely above zero. Different relative importance can be assigned to the two possible case-management errors (failing to treat a patient with malaria and treating a patient without malaria unnecessarily). Although this additional weighting is subjective, it is at least explicit, and can easily be changed to simulate different scenarios.

**Figure 2:**
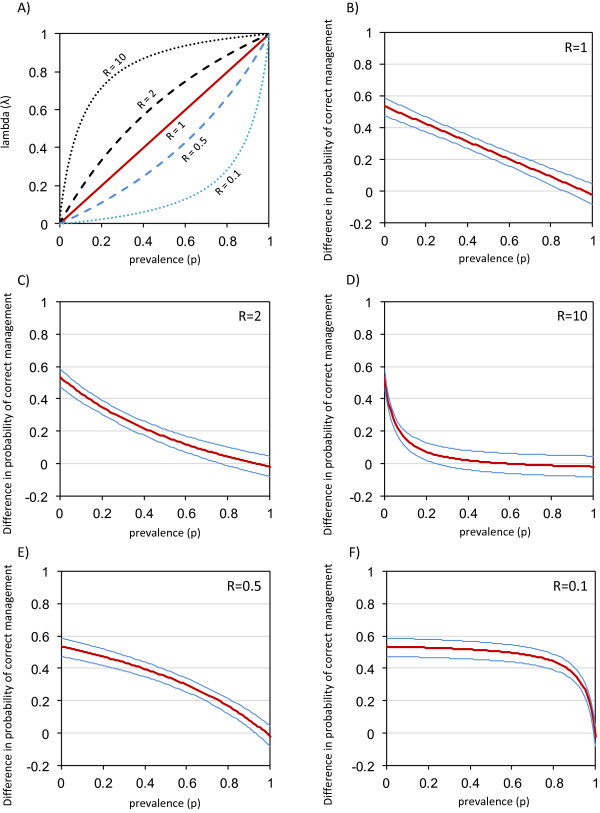
**Illustration of the Newcombe method.** Method from Newcombe [13], using the spreadsheet available from http://medicine.cf.ac.uk/primary-care-public-health/resources/. In [13], the difference in probability of correct diagnosis is plotted against lambda. Here the relationship of lambda to prevalence is shown in figure **A)**, and the difference in probability of correct management is shown against prevalence in figures **B)-F)** for different values of R; blue lines show 95% confidence interval. R is the ratio of importance of false negatives (c1) to the importance of false positives (c2), i.e. R = c1/c2 (Table 4). At prevalence = 0 the difference is equal to the difference in the NMR between the enhanced and control groups, and at prevalence = 1 the difference is equal to the difference in PMR between the two strategies.

The weighted difference in probability of correct management between the two strategies, denoted *f*, is calculated as described in [13] and summarised in Table 4. A plot of the weight, denoted by lambda (λ), against prevalence for five different scenarios is shown in Figure 2A): equal importance given to both types of error (relative importance of false negatives to false positives, R = 1), false negatives considered twice as important as false positives (R = 2), false negatives ten times as important as false positives (R = 10), false positives twice as important as false negatives (R = 0.5), false positives ten times as important as false negatives (R = 0.1). This shows that if the PMR is prioritised (R > 1), i.e. false negatives (missed malaria cases) are emphasised, lambda rises more steeply at low prevalence, and thus the PMR has more influence on the weighted difference. Conversely, if the false positives are prioritised (R < 1), then the NMR has more influence.

**Table 4:** **Mathematical details of the Newcombe method**

1.	For each value of prevalence (p) from 0 to 1, the difference in probability of correct management between the strategies, denoted *f* , is obtained by calculating a (weighted) mean of the difference in the PMR between the two strategies (θ_1_) and the difference in the NMRs (θ_2_):
	*f* = λθ_1_ + (1 - λ)θ_2_
	where the weight, λ is calculated as and
	c_1_ = subjective weight indicating ‘importance’ of treating a malaria patient (avoiding false negatives)
	c_2_ = subjective weight indicating ‘importance’ of avoiding treating a patient without malaria (false positives)
2.	For clarity of presentation the relative importance of false negatives to false positives is defined as
	R = c_1_/c_2_
	If c1 = c2 (R = 1) the equation for λ simplies to i.e. λ = p
3.	A confidence interval for *f* can be calculated as described in Newcombe [13] and http://medicine.cf.ac.uk/primary-care-public-health/resources/.

The implications of this for the weighted difference in probability of correct management between the two strategies are shown in Figures 2B-F, using data from the study by Mbacham *et al.* [19] as an example. Where the confidence interval for the weighted mean crosses 0, this indicates that the experimental group is no longer significantly better than the control group. Assuming equal weight of missed malaria patients and unnecessary treatments, there is an advantage of the enhanced training group in all situations apart from very high prevalence (Figure 2B), due to the increased specificity. If false negatives are considered twice as important (Figure 2C), this reduces the upper prevalence limit where the enhanced group would be considered significantly superior to around 70%. If missed malaria cases are considered to be ten times more important than treated negatives (Figure 2D), this prevalence limit is around 20%, because the slightly lower PMR in the enhanced training group is more heavily penalised. Figure 2E and 2F show that if specificity is considered more important (which is unlikely to be the case for malaria, but shown for completeness), the better NMR offered in the enhanced group means it is superior at all but the very highest levels of prevalence. This approach therefore provides a simple visual way to summarise the two aspects of case management performance, and extend results from one setting to areas with different prevalence. A limitation of this simple graphical approach is that adjustment is not made for covariates, but regression-based approaches to do this are available [20]. Extending results between settings is also complicated by the fact that the prior belief of clinicians and health workers that a patient has malaria will vary between settings with different current (or historic) malaria prevalence. This may influence the success of a specific intervention package intended to improve case management on the basis of RDTs.

## Conclusions

For the reasons outlined above, analysis of case management stratified on positives and negatives will usually be more informative than an analysis that combines correctly managed positives and negatives into a single ‘success’. Stratified results are more comparable between settings, and are therefore more useful to others working in areas where prevalence is different, or where prevalence is changing. An implicit requirement for assessing case management according to parasitaemia status is that a test result is available for each person managed; this requires that a test is available and that the health worker chooses to use it. In practice, this will not always be the case. Understanding what proportion of individuals that should be tested are not tested, and why, could be done separately, with the approaches discussed above then used for those with a test result.

Having tested for malaria, one result gives a straightforward course of action: parasitaemic individuals with fever or other symptoms of malaria should receive an ACT. For the individuals seeking treatment who do not have malaria, defining an appropriate course of action is more complex. One point is that they do not need to receive ACT, but assessing appropriate treatment will require additional clinical judgement and potentially use of further diagnostics, since having established that malaria is not the cause of illness is only a first step in ensuring that the true causes are identified and the patient appropriately managed [7]. Reporting a single metric combines these two distinct issues and is therefore unlikely to be helpful in terms of advancing management of either malaria or febrile illness of other aetiologies. Separate presentation and analysis of patients with malaria and patients without malaria should therefore be encouraged. Where a summary measure is needed, approaches from the diagnostic literature are likely to be more appropriate than a simple combined outcome, although caution is needed in interpretation, and, ideally, overall clinical outcomes should be evaluated.

## Abbreviations

ACT: Artemisin-based combination therapy
CMR: Combined management rate
DOR: Diagnostic odds ratio
LR^+^: Likelihood ratio for positives
LR^-^: Likelihood ratio for negatives
NMR: Negative management rate
PMR: Positive management rate
RDT: Rapid diagnostic test.
